# The dietary management of calcium and phosphate in children with CKD stages 2-5 and on dialysis—clinical practice recommendation from the Pediatric Renal Nutrition Taskforce

**DOI:** 10.1007/s00467-019-04370-z

**Published:** 2019-10-30

**Authors:** Louise McAlister, Pearl Pugh, Laurence Greenbaum, Dieter Haffner, Lesley Rees, Caroline Anderson, An Desloovere, Christina Nelms, Michiel Oosterveld, Fabio Paglialonga, Nonnie Polderman, Leila Qizalbash, José Renken-Terhaerdt, Jetta Tuokkola, Bradley Warady, Johan Vande Walle, Vanessa Shaw, Rukshana Shroff

**Affiliations:** 1 Great Ormond Street Hospital for Children NHS Foundation Trust, and University College London, Institute of Child Health, WC1N 3JH, London, UK; 2 Nottingham Children’s Hospital, Nottingham University Hospitals NHS Trust, Nottingham, UK; 3 Emory University and Children’s Healthcare of Atlanta, Atlanta, USA; 4 Children’s Hospital, Hannover Medical School, Hannover, Germany; 5 Southampton Children’s Hospital, University Hospital Southampton NHS Foundation Trust, Southampton, UK; 6 University Hospital Ghent, Ghent, Belgium; 7 PedsFeeds LLC, University of Nebraska, Nebraska, USA; 8 Emma Children’s Hospital, Amsterdam University Medical Center, Amsterdam, The Netherlands; 9 Fondazione IRCCS Ca’Granda Ospedale Maggiore Policlinico, Milan, Italy; 10 British Columbia Children’s Hospital, Vancover, Canada; 11 Great Northern Children’s Hospital, Newcastle upon Tyne, UK; 12 Wilhelmina Children’s Hospital, University Medical Center Utrecht, Utrecht, The Netherlands; 13 Children’s Hospital and Clinical Nutrition Unit, University of Helsinki and Helsinki University Hospital, Helsinki, Finland; 14 Children’s Mercy Kansas City, Kansas City, USA; 15 University of Plymouth and University College London Institute of Child Health, London, UK

**Keywords:** Calcium, Phosphate, Nutrition, Chronic kidney disease (CKD), Children

## Abstract

In children with chronic kidney disease (CKD), optimal control of bone and mineral homeostasis is essential, not only for the prevention of debilitating skeletal complications and achieving adequate growth but also for preventing vascular calcification and cardiovascular disease. Complications of mineral bone disease (MBD) are common and contribute to the high morbidity and mortality seen in children with CKD. Although several studies describe the prevalence of abnormal calcium, phosphate, parathyroid hormone, and vitamin D levels as well as associated clinical and radiological complications and their medical management, little is known about the dietary requirements and management of calcium (Ca) and phosphate (P) in children with CKD. The Pediatric Renal Nutrition Taskforce (PRNT) is an international team of pediatric renal dietitians and pediatric nephrologists, who develop clinical practice recommendations (CPRs) for the nutritional management of various aspects of renal disease management in children. We present CPRs for the dietary intake of Ca and P in children with CKD stages 2–5 and on dialysis (CKD2-5D), describing the common Ca- and P-containing foods, the assessment of dietary Ca and P intake, requirements for Ca and P in healthy children and necessary modifications for children with CKD2-5D, and dietary management of hypo- and hypercalcemia and hyperphosphatemia. The statements have been graded, and statements with a low grade or those that are opinion-based must be carefully considered and adapted to individual patient needs based on the clinical judgment of the treating physician and dietitian. These CPRs will be regularly audited and updated by the PRNT.

**Electronic supplementary material:**

The online version of this article (10.1007/s00467-019-04370-z) contains supplementary material, which is available to authorized users.

## Introduction

The provision of adequate calcium (Ca) and phosphate (P) is an important part of chronic kidney disease (CKD) management [1]. A low Ca or P intake may lead to poor bone mineralization, a hallmark of mineral and bone disorder associated with CKD (CKD-MBD) [2, 3]. However, excess intake of Ca and P is also detrimental, and may lead to nephrocalcinosis and vascular calcification [4].

The Pediatric Renal Nutrition Taskforce (PRNT) is a team of pediatric renal dietitians and pediatric nephrologists from eight countries in Europe and North America, and endorsed by the International Pediatric Nephrology Association (IPNA) and the European Society for Paediatric Nephrology (ESPN). Dietary management in CKD is an area fraught with uncertainties, with wide variations in practice, and where expert dietetic input, even in tertiary pediatric nephrology centers, is often lacking.

The PRNT recognizes that there are few studies in children with CKD that provide high-level evidence and have undertaken a process of developing clinical practice recommendations (CPRs) for their nutritional management. Existing guidelines on the nutritional requirements of healthy children of all ages were reviewed and requirements for children with CKD proposed. Our CPRs are based on an in-depth review of the available evidence, but in the absence of applicable studies, guidance is based on the opinion of experienced dietitians and nephrologists from the PRNT. These recommendations are designed to provide information and assist in decision-making in order to reduce uncertainty and improve patient outcome. They are not intended to define a standard of care and should not be interpreted as an exclusive course of management. These CPRs will be audited and revised periodically by the PRNT. We also provide research recommendations.

Following guideline publication the PRNT have planned a dissemination phase to guide practical day-to-day management of calcium and phosphate intake. This will involve developing material (written information in the form of leaflets and charts, mobile phone Apps, videos, etc.) both for the child (age-appropriate format) and their parents or carers as well as for health care professionals. Regional variations in diet will be addressed. Translation of this information into different languages will also be performed.

## Methods

The development process for the CPRs, including the group composition and task distribution, is described in the Supplementary material.

### Developing the PICO questions

Recommendations in CPRs are most useful when they provide specific actionable advice on choosing between alternative approaches in particular clinical situations [5]. We developed clinical questions to be addressed by each statement and framed them in a searchable format, with specification of the patient group (P) to whom the statement would apply; the intervention (I) being considered; the comparator (C) (which may be “no action” or an alternative intervention); and the outcomes (O) affected by the intervention [5]. Our PICO terms were as follows:

- Population: Children from birth to 18 years of age with CKD2-5D
- Intervention: Nutritional requirements for Ca and P in children at different stages of CKD
- Comparator: Nutritional requirements for Ca and P in age-matched healthy controls
- Outcomes: Growth, bone disease, fracture risk, Ca balance, bone mineralization on imaging or biopsies, development of hypo- or hypercalcemia, hypo- or hyper-phosphatemia or hyperparathyroidism, and development of vascular calcification

The choice of P binder treatment is not within the scope of this document. The management of hyperphosphatemia in childhood CKD is covered in the Kidney Disease Improving Global Outcomes (KDIGO) 2017 CKD-MBD update [6] and the UK based National Institute for Health and Care Excellence (NICE) guideline on hyperphosphatemia management (published in 2013 and updated in July 2017) [7, 8]. We have not discussed dietary sources of vitamin D as natural (non-fortified) foods provide only a negligible amount of vitamin D [9] and do not significantly alter the serum levels of 25OHD or Ca [10].

### Literature search

Details on the literature search are described in Supplemental Table 1.

### Framing advice

After critically reviewing the literature for each question, we derived CPRs and graded them as suggested by the American Academy of Pediatrics (Supplemental Table 2) [11]. The strength of a recommendation is graded as strong, moderate, weak, or discretionary (when no recommendation can be made). The quality of evidence is graded high (A), moderate (B), low (C), or very low (D). Grade X refers to exceptional situations where validating studies cannot be performed and benefit or harm clearly predominate: in that case, a moderate or a strong recommendation may be given.

Using the Delphi method, voting group members were sent an e-questionnaire to provide a level of agreement on a 5-point scale (strongly disagree, disagree, neither agree nor disagree, agree, strongly agree) and given the opportunity for re-wording of recommendations if appropriate. Participants for the Delphi survey were selected based on the following three criteria: (i) a registered doctor or dietitian currently practising in pediatric nephrology; (ii) at least 5 years' experience with children and young people with CKD2-5D; (iii) currently associated with a major pediatric renal centre. It was agreed *a priori* that at least a 70% level of consensus was required for each statement, failing which the recommendation statement would be adapted after discussion in the core group, and reviewed again by the voting panel until a consensus level of at least 70% was achieved.

## Clinical Practice Recommendations

1. What are the main dietary sources of Ca and P for an infant, child and adolescent?
  1.1 The main dietary sources of Ca for children are milk, milk products, breast milk and manufactured infant formulas. Statutory or voluntary fortification of foods with Ca can increase the contribution from other foods. (ungraded)
  1.2 The main natural dietary sources of P for children are milk (including milk products, breast milk and manufactured infant formulas), cereal (grains) and cereal products, and meat and meat products. Inorganic P added to some processed foods is readily absorbed and can significantly increase P intake. (ungraded)

### Evidence and rationale

#### Dietary sources of calcium

The contribution of foods to average daily intake of Ca at different ages is presented in Table 1. International data suggest that dairy products are the largest contributor to dietary Ca intake in most children, providing 69% of Ca intake in Dutch children [14], 44–70% of Ca intake in UK children [12, 13], 49% of Ca intake in French children and adolescents (3–17 years) [15], and 37% of Ca intake (for all age groups) in the USA [16]. In early infancy, breast milk or infant formulas comprise 100% of the daily dietary Ca intake. All standard and most specialized infant formulas have a higher concentration of Ca relative to human milk, but lower than cow’s milk or formulas intended for older children. Formulas designed for infants must comply with set compositional criteria, providing a minimum amount of 50–60 mg Ca per 100 kilocalories and a Ca:P ratio between 1.1 and 2.0 [17, 18]. The typical Ca content per portion of Ca-rich foods is shown in Table 2. The Ca content of reduced-fat dairy products is comparable to whole milk–derived products. Hard water from taps and some mineral waters can be important sources of Ca [19, 20], with a bioavailability similar to dairy products [21].

**Table 1:** Percentage contribution of food types to average daily intake of calcium (Ca)

% Total dietary Ca intake							
Food group	Age (years)						
	1.5–2.5	2.5–3.5	3.5–4.5	4–6	7–10	11–14	15–18
Cereal (grain) and cereal products	16	20	22	23	27	28	27
Milk and milk products	70	63	59	55	48	45	44
Eggs and egg dishes	1	1	1	1	1	1	1
Meat and meat products	2	3	3	4	6	6	7
Fish and fish dishes	1	1	1	2	2	1	2
Vegetables, potatoes and savory snacks	3	3	4	5	5	6	7
Fruit and nuts	1	1	1	1	1	1	1
Sugars, preserves and confectionary	3	3	4	3	4	5	4

Adapted from National Diet and Nutrition Survey (1995 and 2000) [12, 13]

**Table 2:** A guide to the calcium (Ca) content of various food groups

Food	Portion size	Ca mg per portion
Dairy and dairy products		
Human breast milk (mature)	100 ml	34
Standard whey based infant formula	100 ml	55
Cow’s milk	100 ml	120
Fortified oat, hemp, coconut, rice and almond milk	100 ml	120
Custard or rice pudding	120 g	170
Hard cheese	30 g	240
Soft cheese (e.g., brie, mozzarella)	30 g	120
Yoghurt	80 g (small pot)	90
Dairy free yoghurt*	125 g	130
Egg		
Egg, cooked	50 g (1 egg)	28
Soya products		
Soya milk, cheese and desserts*	Check individual product for degree of calcium fortification	
Calcium-set tofu**	50 g (2 tablespoons)	60
Fortified orange juice		
Calcium fortified orange juice	100 ml	120
Cereal (grain) and cereal products*		
Bread - white fortified	33 g slice	58
Bread - wholemeal	33 g slice	35
Fortified breakfast cereals	30 g portion	80–146
Fruit		
Apricots, raw	4	45
Figs, dried/ready to eat	5	230
Currants	2 tablespoons	50
Orange	1	75
Fish (soft bones eaten)		
Anchovies, canned	½ small tin	75
Sardines (tinned in oil)	1 sardine	125
Whitebait, fried	40 g	430
Salmon, tinned	100 g	90
Nuts and seeds		
Almonds/brazil nuts/hazelnuts/walnuts	6–20	30–60
Sesame seeds	1 tablespoon	65
Spreads		
Peanut butter	2 tablespoons	20
Almond butter	1 tablespoon	40
Hummus	100 g (½ tub)	45
Tahini paste	1 heaped teaspoon	125
Vegetables		
Broccoli	3 florets	35
Watercress	20 g	35
Curly kale	100 g	150
Okra, stir fried	8	90
Chickpeas	3 tablespoons	45
Red kidney beans	3 tablespoons	40
Green beans	90 g	50
Baked beans	150 g (small tin)	80

*Ca fortification of foods such as bread, breakfast cereals, milk alternatives (including plant based drinks), and fruit and vegetable drinks is practised in some countries. It is advised to refer to country specific food composition tables where possible

**Tofu can be a good source of calcium as it is made by coagulating soymilk with salts such as calcium sulfate or magnesium chloride—the levels of calcium are dependent on the coagulant used; we have called this calcium-set tofu. The Ca and phosphate content in various tofu products may be similar to cow’s milk (i.e., 120 mg/100 g), but can vary widely. Refer to product-specific values

The National Diet and Nutrition Survey (NDNS) [12, 13], a continuous cross-sectional survey of a representative sample from the UK general population above 18 months of age, is the most recent detailed data for children describing the relative contribution of different food groups to dietary Ca intake (Table 1). The “milk and milk products” food group (includes milk, cheese, yogurt, cream, and ice cream) contributes 44–70% of dietary Ca intake, being highest in the younger age groups. “Cereal and cereal products” (comprising grains made into pasta, rice, breads, breakfast cereal, and biscuits) contribute 16–28% of average daily Ca intake. This mainly reflects the contribution from Ca-fortified flour in cereals in the UK, particularly in older children [22]. Other sources of Ca, including vegetables, fish, meat, fruit, and confectionary, each contribute between 1 and 7% of daily Ca intake (see Table 1). The variation in foods eaten in different countries or cultures may alter the relative Ca intake from specific food groups. The most recent NDNS reports that the average dietary Ca intake has significantly decreased in young children (aged 1.5 months to 10 years) from 2008 to 2017 [23].

Calcium may be added to foods, either for fortification or as a food additive. Some countries mandate Ca fortification of foods, such as Ca fortification of bread and wheat flour (except wholemeal) in the UK [24]. Ca is also added voluntarily by manufacturers to some breakfast cereals and drinks to increase the Ca content. Ca-containing additives (used as food colors, preservatives, and antioxidants rather than for the purpose of nutritional fortification) are frequently used in processed foods and can contribute 170–430 mg of Ca per day or between 15 and 30% of Ca intake depending on the child’s age [25]. Nutrient composition tables include the contribution from naturally occurring Ca and some fortified products (e.g., bread and breakfast cereals), but the actual content of processed products vary depending on the production methods, recipe, and brand. However, the calculated estimates of dietary Ca intake using nutrient databases and dietary assessment software packages are considered reasonably accurate when compared to direct chemical analyses [26]. There are international recommendations on Ca intake for healthy children (Table 6).

#### Dietary sources of phosphate

Dietary P is available in two forms, organic and inorganic, with inorganic phosphates added during food processing. The UK NDNS reports that “milk and milk products” and “cereal and cereal products” are the main sources of dietary P intake, contributing 23–35% and 24–27% of total intake, respectively [12, 13]. Data from the USA for all age groups also indicate that the highest contributors to P intake are milk, meat, and grains [27, 28]. As with Ca intake, the highest percentage intake of P comes from “milk and milk products,” particularly in the younger age groups, while “meat and meat products” account for 15–20% of P intake, being highest in older children. Breast milk is relatively low in phosphate, having a concentration about half of most standard whey-based formulas and one-third of casein-based infant formulas. The minimum level (25–30 mg per 100 kilocalories), but not maximum level, of phosphate in standard infant formulas is regulated [17, 18]. The relative contribution of foods to average daily intake of P at different ages in the UK is presented in Table 3, but data on food P content are often incomplete due to the inability to accurately quantify the contribution from P additives. Table 4 shows the typical P content per portion of foods with high P content.

**Table 3:** Percentage contribution of food types to average daily intake of phosphate (P)

% Total dietary P intake				
Food group	Age (years)			
	4–6	7–10	11–14	15–18
Cereal (grain) and cereal products	24	27	26	24
Milk and milk products	35	29	25	23
Eggs and egg dishes	1	2	2	2
Meat and meat products	15	17	19	20
Fish and fish dishes	3	3	3	3
Vegetables, potatoes, and savory snacks	11	12	13	14
Fruit and nuts	2	2	1	1
Sugars, preserves, and confectionary	3	3	4	3

Adapted from National Diet and Nutrition Survey (1995 and 2000)

**Table 4:** A guide to the phosphate (P) content of various food groups

Food	Portion size	Phosphate mg per portion	Phosphate additives^#^
Dairy and dairy products			
Human breast milk (mature)	100 ml	15	
Standard whey based infant formula	100 ml	32	
Cow’s milk	100 ml	100	−
Yoghurt	125 g	100–200	−/++
Fromage frais	60 g	70	−/+
Ice cream	100 g (2 scoops)	100	−/+
Cheese, hard (cheddar, edam, gouda, emmental)	1 thin slice (25 g)	120–160	++
Cheese, soft (camembert, mozzarella)	30 g portion	80	−/+
Processed cheese	25 g	250	+++ (high bioavailability)
Cottage cheese	1 tablespoon (40 g)	50–70	−
Egg			
Egg	50 g (1 egg)	100	−
Egg white	30 g (from 1 egg)	4	
Soya products			
Soya milk (not calcium-enriched)	100 ml	10-50	−
Soya milk (calcium-enriched)	100 ml	50-100	−
Tofu (depending on production and cooking method)	2 tablespoons (50 g)	50–135	−
Meat and meat products			
Lamb, pork, beef, fish, burgers, chicken	100	130-220	++
Beefburger	1	100	++ (high bioavailability)
Beef mince	3 tablespoons (75 g)	100	++
Sausage	1 (or 2 chipolatas)	100	++
Chicken-drumstick	1	100	−/+
- Breast	½	100	−/+
- Nuggets	6	100	++
Cold meat (ham, chicken roll)	1 slice (25 g)	80	++ (high bioavailability)
Fish filet (small)	50 g	100	−/+
Fish fingers	2		−/+
Prawns	10		−/+
Salmon	1/3 salmon steak		−/+ (canned products)
Scampi	3 pieces		−/+
Pulses (beans/lentils) and nuts			
Baked beans	2 tablespoons (80 g)	70	−/+ low bioavailability)
Nuts	1 small bag (25 g)	120	−/+ (low bioavailability)
Dahl	2 tablespoons (80 g)	60	−/+ (low bioavailability)
Cereal (grain) and cereal products			
Bread - white	1 slice (30 g)	30	−/+ (raising agent)
Bread - wholemeal	1 slice (30 g )	60	−/+ (raising agent)
Bran type breakfast cereals	1 small bowl (30 g)	100–200	−/+
Wheat based breakfast cereals (wheat biscuits/cookies)	1 biscuit/cookie (20 g)	50	−/+
Confectionary and drinks			
Milk chocolate	1 bar (50 g)	110	−/+
Chocolate covered biscuit/cookie	1 biscuit/cookie (18–22 g)	20–40	−/+
Cola drink	1 can (330 ml)	100	++ (high bioavailability)

^#^–, no added phosphate; –/+, added phosphate in some products; +, added phosphate in most products; ++, large quantities of added phosphate in most products

Table adapted from Ritz et al, 2012 [31] and Kalantar-Zadeh et al, 2010 [27]

Phosphate additives, such as phosphoric acid and sodium phosphate, are used by the food industry to improve the texture, taste, appearance, and shelf-life of many processed foods and their presence, but not quantity, may be indicated on the food ingredient list as an E number (Table 5). P additives may increase the P content by 60–70 mg per 100 g of product [29]. Manufacturers are not legally required to list the total P content on the food label or the contribution to daily P requirements, despite increasing pressure for this to be mandatory [30]. Processed foods with significant amounts of added inorganic P include meat, dairy, and bakery products, as well as a growing number of drinks such as colas and some flavored drinks, bottled water, malted drinks and sterilized, ultra-heat treated, and thickened or powdered milks [31, 32] (Table 4). In addition, inorganic phosphates are added to several medications, such as antacids and many anti-hypertensive medications, as an excipient that aids in dispersion of the active drug ingredient once ingested [33, 34]; these can vary between manufacturers and across different formulations of the same medication. The P load from medications can make a meaningful contribution to the daily P intake, and thus should be reviewed carefully, particularly in patients receiving anti-hypertensive medications. Nutrient databases and dietary assessment software packages are used to assess P intake, but they do not include the amount of inorganic P derived from P additives. Consequently, assessed total daily P intake in individual and national dietary surveys may underestimate the true P intake by over 20% [26, 30, 35]. This is especially relevant for children, who may have a high intake of processed foods [31]. There are international recommendations on P intake in healthy children (Table 6).

**Table 5:** Phosphate (P) containing EU approved additives commonly used in Europe

E 338 Phosphoric acid (acidifier in colas and jams) E 339 Sodium phosphates (emulsifier in processed cheese) E 340 Potassium phosphates (stabilizer and thickener in processed meats) E 341 Calcium phosphates (leavening agent in baked goods) E 343 Magnesium phosphates (antacid) E 450 Diphosphates (emulsifier and stabilizer in flour) E 451 Triphosphates (preservative in canned products)	E 452 Polyphosphates (quality enhancer for meat and fish) E 541 Sodium aluminum phosphates (chemical leavening of baked goods) E1410 Monostarch phosphate (thickeners and stabilizers in foods such as puddings, custards, soups, sauces, gravies, pie fillings, and salad dressings) E1412 Distarch phosphate (stabilizes the consistency of the foodstuff when frozen and thawed) E1413 Phosphated distarch phosphate (stabilizes the consistency of the foodstuff when frozen and thawed) E1414 Aceylated distarch phosphate (gluten free and can be used as a stabilizer, thickener, binder or emulsifier) E1442 Hydroxyl propyl distarch phosphate (thickening, and texturing agent in food products provides greater shelf life, enhances shine and color to products and has excellent cold storage properties)

*The numbering scheme for food additives follows the International Numbering System (INS) as determined by Codex Alimentarius, the international food standards organization of the World Health Organization (WHO) and Food and Agriculture Organization (FAO) of the United Nations (UN). Only a subset of INS additives are approved for use in the European Union as food additives. The USA does not follow the INS system. The inclusion of “phos” as part of an ingredient on the food label in North America indicates the presence of phosphate

**Table 6:** International recommendations for calcium and phosphate in healthy children

Calcium					Phosphate			
	EFSA (2015)	D-A-C-H (2015)	NCM (2014)	IOM (2011)	EFSA (2015)	D-A-C-H (2015)	NCM (2014)	IOM (1997)
Age (months)	–	0 - < 4	0 < 6	0 < 6	–	0 - < 4	0 < 6	0 < 6
PRI or RDA (mg/day)		220	– BF only	200 (AI)		120	–	100 (AI)
Age (months)	7–11	4 - < 12	6-11	6–12	7–11	4 - < 12	6–11	7–12
PRI or RDA (mg/day)	280 (AI)	330	540	260 (AI)	AI 160	300	420	275 (RDA)
Age (years)	1–3	1 - < 4	1-5	1-3	1-3	1 < 4	1–5	1–3
PRI or RDA (mg/day)	450	600	600	700 (RDA)	250	500	470	460 (RDA)
Age (years)	4–10	4 - < 7	6-9	4-8	4–10	4 - < 7	6–9	4-8
PRI or RDA (mg/day)	800	750	700	1000 (RDA)	440	600	540	500 (RDA)
Age (years)	11–17	7 - < 10	10–17	9–18	11–17	7 - < 10	10–17	9–18
PRI or RDA (mg/day)	1150	900	900	1300 (RDA)	640	800	700	1250 (RDA)
Age (years)	18-24	10 - < 13			18 - 24	10 - < 19		
PRI or RDA (mg/day)	1000	1100			550	1250		
Age (years)		13 - < 19						
PRI or RDA (mg/day)		1200						

EFSA, European Food Safety Authority; D-A-C-H, Deutschland-Austria-Confoederatio Helvetica; NCM, Nordic Council of Medicine; IOM, Institute of Medicine; PRI, Population Reference Intake; RDA, Recommended Dietary Allowance BF, breast fed. PRI and RDA are terms used to reflect the amount of a nutrient that is likely to meet the needs of almost all (97.5%) healthy people in a population or the average amount plus two standard deviations (assuming individual requirements are normally distributed with a population). If the average intake of an otherwise healthy individual (or population) is at or above the PRI or RDA, then the risk of deficiency is judged to be very low. However, if the average regular intake is below this then it is likely that some will have an intake that may be insufficient. AI (used by EFSA and IOM) is a dietary recommendation used when there is not enough data to calculate an average requirement. An AI is the average nutrient level consumed daily by a typical healthy population, which is assumed to be adequate for the population's needs

#### Bioavailability of dietary Ca and P

The absorption of Ca from foods and medications (Ca-based P-binders and Ca supplements) is about 30%, but varies between 5 and 82% depending on the food source and subject-related factors [36], particularly their vitamin D status. The Ca from breast milk and infant formula has an absorption efficiency of about 66% and 40%, respectively. However, as formulas have a higher concentration of Ca than breast milk, this comparison in bioavailability may not be important [37], and absorption values similar to breast milk have been reported for some specialized formulas [38]. Milk, dairy products, and fortified foods have a Ca bioavailability between 30 and 40%, whereas it is below 10% for vegetables and fruit (such as spinach and rhubarb). Thus, although some cereals and green leafy vegetables and fruit may be rich in Ca (and also low in phosphate), given the low bioavailability of Ca from these sources, children with CKD are unlikely to consume sufficient quantities in order to meet nutritional requirements. The efficiency of absorption is influenced by the oxalate and phytic acid content of the food, the amount consumed [32], vitamin D status [39], and the child’s age. In patients with vitamin D deficiency, defined as a serum 25-hydroxyvitamin D level less than 75 nmol/L [10], only 10–15% of dietary Ca and approximately 60% of dietary P is absorbed, while the efficacy of intestinal Ca and P absorption in vitamin D replete subjects increases to 30–40% and 80%, respectively [9, 40]. The amount of Ca absorbed from P-binders is not fully understood [41].

The absorption of P from foods depends on whether it is in an organic or inorganic form, and on vitamin D status. Meats, fish, dairy, vegetables, grains, and nuts contain organic carbon-bound P, with a bioavailability between 30 and 70% [42–44]. Plant-based organic P (including seeds and legumes) is stored in the form of phytate or phytic acid, which cannot be broken down by humans, reducing bioavailability to 30–40% [45]. However, inorganic P salts added to processed foods are not organically bound or associated with phytate, resulting in a bioavailability of up to 100% [35, 46, 47]. Given that children often consume processed foods, the potential for high intake of bioavailable inorganic P additives is significant. A list of P-containing food additives approved in Europe [48] are presented in Table 5. The food additives approved in the USA are available on the FDA website [49].

Foods with comparable P loads may contribute significantly different nutritional values. For example, both a whole cooked egg and a can of cola-based drink contain approximately 80 mg phosphate. However, the nutrient-rich egg provides an excellent source of protein, fat, B-vitamins, and selenium, compared to the nutrient-poor empty-calories in the cola. The egg’s organic phosphate load has a low bioavailability, yielding only 40% (36 mg) absorption, compared to cola’s highly bioavailable inorganic phosphate, yielding 100% (80 mg) absorption.

2. How are Ca and P intake assessed in healthy children and children with CKD2-5D?

We suggest that in healthy children and those with CKD2-5D a diet history of a typical 24-h period be used to rapidly identify the main dietary sources of Ca and P, including P additives in processed foods. A 3-day prospective diet diary/food intake record may be used when detailed information is required. An estimate of the total Ca and P intake should consider contributions from diet, nutritional supplements, dialysate, and medications, including P binders (grade C, weak recommendation).

### Evidence and rationale

An estimate of usual Ca and P intake can be made using a variety of tools (Supplemental Table 3), each with advantages and disadvantages. Many of these have been developed for research purposes or for particular population groups, and vary in their accuracy as well as respondent and interviewer burden [50, 51]. Either a 3-day prospective diet diary/food intake record (semi-quantitative or weighed) or a food frequency questionnaire (FFQ) can give a clinically useful estimate of intake [52]. The 3-day prospective diet diary/food intake record is considered “gold standard” against which other dietary assessment methods are compared, but can be time consuming to administer and assess. FFQs tend to overestimate daily dietary nutrient intake, although this depends on the number of food categories included in the tool [53–55]. Supplemental Table 3 lists the relative advantages and disadvantages of the different methods of dietary assessment.

Clinicians may prefer to rapidly estimate calcium intake from a retrospective diet history of a typical 24-h period. This is a detailed dietary assessment technique consisting of questions about food and drinks consumed at meals and snacks through a typical 24-h period. This usually requires about 20–30 minutes by a dietitian or suitably trained designate. The history is usually taken from morning to night, capturing information on usual meal pattern and types, frequency, and quantity of foods consumed. Any additional nutrient source (e.g., nutritional supplements) should be included. Tools that can assist in estimation of portion sizes include use of common household measures (e.g., teaspoons, tablespoons, slices, and handfuls), food portion booklets [56], and photographic representations of portion sizes. A more detailed account of the frequency of consumption of specific dietary sources of Ca or P (such as milk, cereals, and additive-rich foods) can be ascertained by direct questioning. An estimate of Ca or P intake can be made from the diet history by reference to guides of the Ca and P content of common food items (see Tables 2 and 4). If a more accurate assessment is required, the portion sizes for each dietary item can be converted into weights and used with country specific food composition databases (FCDB) or dietary analysis software, supplemented by other sources, such as information from manufacturers, if required. The frequency of assessing the dietary intake should be guided by the child’s age, CKD stage, relevant nutritional concerns, or the presence of abnormal serum biochemistry.

Importantly, the Ca intake from P binders can contribute significantly to dietary Ca intake and must be included when calculating total daily Ca intake in CKD patients. There is little data on the Ca absorption from binders, particularly the effects of age and residual kidney function. Ca carbonate contains 40% Ca with an absorption of 20–30% and Ca acetate contains 25% Ca with an absorption of 20% when taken with meals [57, 58].

3. What are the Ca and P requirements in an infant, child and adolescent?
  3.1 Requirements in healthy children

We describe the Ca and P requirements for healthy children as background and justification for estimating the requirements for children with CKD2-5D; specific recommendations for healthy children are outside the scope of this document.

### Evidence and rationale

Adequate dietary Ca intake during childhood and adolescence is essential for normal skeletal mineralization [59]. The Ca content of the skeleton increases from 25 g at birth to around 1000 g in an adult female and 1200 g in an adult male. Approximately 25% of the total skeletal mass is laid down during the 2-year interval of peak height velocity during adolescence [60]. The normal levels of serum Ca and P have been previously described [41, 61, 62]. Balance studies indicate that dietary Ca absorption is the main driver of net Ca retention in infants and adolescents [63, 64]. The highest Ca requirements are during periods of rapid growth, including in the first year of life and during puberty, dropping after puberty to normal adult requirements [65]. The higher Ca requirements in young children are associated with higher normal values for serum total Ca, particularly during infancy, and reach adult levels by 5 years of age.

Randomized controlled trials (RCTs) in healthy children have shown that children require a higher Ca intake than adults, presumably for bone mineral accrual and growth [60, 66–68]. A double-blind placebo controlled RCT has shown that in pre-pubescent girls Ca-enriched foods significantly increased bone mass accrual over 1 year, with a preferential effect in the appendicular skeleton [66]. A further RCT has shown that in 12-year-old girls an increase in milk intake led to an increase in bone mineral density (BMD) and bone mineral content as measured by dual-energy x-ray absorptiometry (DXA) scan [67]. A RCT in pubertal girls with up to 7 years of follow-up showed that Ca supplementation significantly influenced bone accretion, particularly during the pubertal growth spurt, and the Ca requirement for growth was associated with skeletal size [68]. In fact, bone mineral accrual continues up to the end of the second or early in the third decade of life, depending on the skeletal site, when peak bone mass is achieved [60].

There is limited data on the Ca intake and health outcomes in normal children or suitable biomarkers of bone mineralization that can be used to derive suggested dietary requirements. An estimate of requirements for healthy infants is based on the Ca and P from breast milk. For children over 1 year of age, most international bodies use the factorial method to assess Ca and P requirements. This method accounts for the total quantity of mineral needed for bone accretion, growth, and replacement of obligatory losses (from stool, urine, and skin), and is adjusted for percentage absorption.

National recommendations for Ca and P intake for normal children have been reported by many countries [69–74] (Table 6). The international publications referenced in Table 6 use the most reliable methods for assessing mineral status. A number of different terms have been used in international recommendations to describe nutrient adequacy; these include Population Reference Intake (PRI), Recommended Dietary Allowance (RDA), Adequate Intake (AI), and others. Since these recommendations for dietary adequacy have different definitions and have used different methods in their derivation, some of the resulting recommendations differ widely. For example, for a 6–11-month-old infant, the Nordic Council of Medicine gives a Recommended Intake for calcium of 540 mg, whereas for a 6–12-month old, the US Institute of Medicine gives an Adequate Intake of 260 mg. We have taken a pragmatic approach and quoted the range of the published values for our recommendations. We refer to this new reference using a novel term, Suggested Dietary Intake (SDI; Table 7). The lower and upper limits of the SDI lie within the international published values for Ca and P intake in healthy children (Table 6), which represent the required average amounts of a nutrient plus 2 standard deviations. Currently, studies of dietary adequacy are not directly comparable because different standards have been used, depending on the publication used as the benchmark. As the SDI encompasses the current published international recommendations, future reference to the SDI as the benchmark for nutritional adequacy will allow better comparison between studies.

**Table 7:** Summary of SDI (suggested dietary intake) for calcium and phosphate in children with CKD2-5D

Age (years)	SDI calcium (mg)	SDI phosphate (mg)
0–< 4 months	220	120
4–< 12 months	330–540	275–420
1–3 years	450–700	250–500
4–10 years	700–1000	440–800
11–17 years	900–1300	640–1250

For children with poor growth, reference to the SDI for height age may be appropriate. This is the age that corresponds to their height when plotted at the 50th centile on a growth chart

3.2 Requirements in children with CKD2-5D
  3.2.1 We suggest that the diet of children with CKD2-5D should be regularly assessed for total Ca and P content. The contribution of P additives to total P intake cannot be quantified, but dietary sources of P additives should be identified where possible. Frequency of assessment is based on the child’s age, CKD stage, and trends in serum Ca, P, and PTH (ungraded).
  3.2.2 We suggest that the total Ca intake from diet and medications, including P binders, should be within the SDI, and be no more than twice the SDI, unless in exceptional circumstances (grade C, weak recommendation).
  3.2.3 In special circumstances, such as for infants with CKD or those with mineral depleted bone, a higher Ca intake may be considered with careful monitoring (grade C, weak recommendation).
  3.2.4 We suggest that the dietary P intake of children with CKD should be within the SDI for age, without compromising adequate nutrition (grade C, weak recommendation).

### Evidence and rationale

As with healthy children, children with CKD require adequate Ca for skeletal mineralization, particularly during periods of active growth. However, excess Ca is potentially detrimental and can lead to ectopic calcification.

Childhood CKD is associated with nearly universal disturbances in bone and mineral metabolism, [2, 6, 75–77] resulting in bone pain, deformities [75, 76], growth retardation [2, 76], and fractures [76, 78]. Abnormal bone micro-architecture and mineralization defects are common and strongly associate with Ca status: mineralization defects were present in 29% of children in CKD2, and nearly 80% in those with CKD4-5 [79]. Similarly, in a cohort of peritoneal dialysis (PD) patients over 90% had deficient bone mineralization [80]. Of 170 children and adolescents with CKD stages 2-5D, 6.5% sustained a fracture over a 1-year follow-up [81], and this associated with lower tibial cortical BMD, particularly in growing children [81], and low serum Ca levels [81]. A prospective cohort study reported that children with pre-dialysis CKD have a 2–3-fold higher fracture risk than their healthy peers [82]. P-binder treatment was associated with a decreased risk of incident fractures, independent of age, sex, eGFR, and PTH levels [82], but of note, 82% of the children who were on P binders received a Ca-based P binder, suggesting that the Ca intake from binders may have a protective effect against fractures.

Conversely, a high Ca intake may lead to vascular calcification [83–85], progressive vessel stiffness [85, 86], left ventricular failure [87], and sudden death [81]. Vascular calcification begins in pre-dialysis CKD [85, 86] and accelerates on dialysis [85, 88]. In young adults on dialysis, the coronary artery calcification score was shown to double within 20 months [83], and worsening of the calcification score was associated with a higher serum Ca-P product and prescription of Ca-containing phosphate binders [83, 84]. Thus, high Ca levels carry a significant risk of vascular calcification and treatment strategies must be carefully tailored to provide enough Ca for mineralization of the growing skeleton, but not too much to cause ectopic calcification and cardiovascular disease.

There are no studies to indicate the appropriate amount of Ca for a child with CKD, and it is very likely that this would need to be individualized depending on the patient’s age, growth, and rate of bone turnover. The main source of Ca in the diet is dairy, which is frequently restricted in CKD due to its high P content. Hence, dietary intake alone may not provide sufficient Ca. Furthermore, intestinal Ca absorption may be impaired due to dysregulation of the normal homeostatic mechanisms. Low levels of activated vitamin D may lead to reduced Ca absorption [9, 40], even in the presence of Ca deficiency [89]. Extrapolation of data from adult CKD studies is not appropriate in this field, as the growing skeleton of children has significantly higher Ca requirements than a mature, possibly osteoporotic, adult skeleton.

In the absence of evidence-based studies, it would be reasonable to provide CKD children with comparable amounts of Ca intake (including Ca from diet, P binders, and supplements) as their healthy peers. Ca intake from all sources should provide at least 100% of the SDI. There are no studies to set a safe upper limit for Ca intake for healthy children of different ages [69]; however, based on expert opinion, KDOQI suggested 200% of the dietary reference intake as a safe upper limit [61]. Thus, the maximum Ca intake we recommend is 2600 mg/day (i.e., 200% of the SDI for 11–17-year olds). This is marginally higher than the KDOQI recommended upper limit of 2500 mg Ca/day as the KDOQI use American DRI, whereas as our SDI is based on recommendations from current international authorities: EFSA, DACH, NCM, and IOM.

Research shows that P retention has a significant role in the development and progression of CKD-MBD, including P-associated vasculopathy [4, 83–85, 90] and defects in bone mineralization [80, 91]. In adults on dialysis, high serum P levels have been linked to vascular calcification and death [92]. Control of serum P is essential for attenuating the progression of secondary hyperparathyroidism and associated cardiovascular damage in children [83–85, 90, 93]. P retention begins early in the course of CKD, although phosphaturic hormones such as fibroblast growth factor 23 (FGF-23) may prevent high serum P levels in early CKD [94, 95]. Nevertheless, it is important to limit dietary P intake to within the SDI in the mild-moderate stages of CKD (except in children with renal tubular disorders) and to the lower limit of the SDI in patients with advanced CKD and persistent hyperphosphatemia or hyperparathyroidism. Whey-based infant formulas have a low P content and continued use of these in place of casein-based formula or cow’s milk beyond infancy may be appropriate. Renal specific formulas may be considered as they have a lower P content, but caution is advised as potassium and Ca intake will also be considerably reduced. Careful advice during transition onto solid foods can control the introduction of higher P foods, especially when the bulk of P is provided from an infant formula. Importantly, aggressive dietary P restriction is difficult since it has the potential to compromise adequate intake of other nutrients, especially protein and Ca; hence, P binders are often required.

4. Managing the Ca and P requirements in children with CKD2-5D
  4.1 We suggest that intake of Ca and P is adjusted to maintain serum Ca and P levels within the age-appropriate normal range, without compromising nutrition. Changes in management should be based on trends of serial results rather than a single result, with integration of serum Ca, P, PTH, alkaline phosphatase, and 25-vitamin D levels (grade C, weak recommendation).
  4.2 We suggest that children with CKD who have hyperphosphatemia or hyperparathyroidism will require further dietary restriction of P, potentially to the lower limit of the SDI, without compromising adequate nutrition. Advice to limit the P contribution from phosphate additives should be given. Use of P binders for further control of serum P and PTH levels is often required, in addition to dietary restriction (grade C, weak recommendation).
  4.3 We suggest that children with persistent hypocalcaemia or a high PTH may require a Ca intake above 200% of the SDI for calcium for short periods and under close medical supervision. Calcium can be provided through Ca supplementation, together with vitamin D (usually both native and active forms), as well as other sources of Ca such as a high Ca dialysate (grade C, weak recommendation).
  4.4 We suggest that children with persistent hypophosphatemia should have their dietary P intake increased. P supplements may be necessary in some patients, particularly those on intensified dialysis or with renal wasting of P (grade C, weak recommendation).

### Evidence and rationale

The intake of Ca and P is titrated based on serum levels of Ca, P, PTH, alkaline phosphatase, and 25-vitamin D considered together, following trends in results rather than single values, and maintaining levels within the age-appropriate normal range. Normal Ca and P levels are age dependent and are higher in infants and younger children [96]. Given the complex inter-dependency of these CKD-MBD measures, it is important to consider trends in levels, particularly for PTH, rather than a single value, as suggested in the KDIGO CKD-MBD recommendations [6]. Ionized Ca is the ideal measure when assessing serum Ca. It is influenced by alterations in albumin, circulating levels of anions, and acid-base status that are common in dialysis patients [97], and thus may not correlate with total serum Ca. Albumin-corrected Ca is superior to uncorrected total Ca, and should be used to modify treatment if ionized Ca is not available.

The intake of Ca from all sources must be considered: diet, medications (including Ca-based P binders and Ca supplements) and dialysate. The Ca intake from the diet may not provide sufficient Ca, particularly as most Ca-rich foods also contain large amounts of P, and are likely to be restricted in CKD patients, as discussed in “Introduction.” Furthermore, as reviewed in “Introduction,” the bioavailability of Ca and P from different foods varies and also depends on the vitamin D status of the individual [9, 10, 40].

The Ca content of P binder medications is shown in Table 8. The indication, dosage, and titration of different P binders, as well as use of different dialysate Ca concentrations, is outside the scope of this guideline and discussed extensively by other guideline groups [6, 7, 61]. It is noteworthy that international guidelines in children recommend the use of Ca-based P-binders as first-line treatment for hyperphosphatemia to help meet their high Ca requirements [41, 57]. However, it is important to consider the wide variation in elemental Ca intake and bioavailability from different Ca salts [61]. In addition, when Ca-containing medications are given with food, the Ca absorption is significantly lower compared to when they are given between meals: Ca absorption from Ca acetate averaged 21 ± 1% when given with food vs. 40 ± 4% when the binder was given while the subject was fasting [58].

**Table 8:** Commonly used phosphate binder medications and their calcium content

Phosphate binder medication	Percentage calcium content	Percentage calcium absorbed when taken with food	Phosphate bound per gram of calcium absorbed (mg/mg)	Comments
Calcium carbonate (commonly available as 250 mg, 500 mg, 1.25 g, 2.5 g tablets)	40	20-30	≈ 1 mg/8 mg	High calcium load; usually well tolerated with few gastrointestinal side-effects; requires an acidic pH in the stomach to dissociate into calcium and carbonate, hence must not be given with antacids or H2-receptor blockers; disperses easily when crushed and added to feeds; inexpensive.
Calcium acetate (available as 475 mg or 950 mg tablets)	25	22	≈ 1 mg/3 mg	Less calcium load than CaCO_3,_; few gastrointestinal side-effects, but may not be well tolerated in infants; forms a suspension when mixed in feeds; can thicken or curdle some feeds; inexpensive.
Mg and Ca carbonate combination tablets (variable tablet strength)	Variable	20-30	≈ 1 mg/2.3 mg	Less calcium load than CaCO_3_ alone; gastrointestinal side-effects including diarrhea from the magnesium content; magnesium may have a protective effect on development of vascular calcification.
Sevelamer hydrochloride (800 mg tablet) or sevelamer carbonate (800 mg tablet or 2400 mg sachet)	0	0	Not applicable	Calcium free; may be difficult to administer in young children; expensive. Tablet is too hard to crush. Cannot be added into feeds, but will form a gel when mixed with water and allowed to stand. Can block feeding tubes if not flushed through.
Lanthanum carbonate (available as chewable tablets or sachets with 500 mg, 750 mg or 1000 mg elemental lanthanum)	0	0	Not applicable	Poorly tolerated as gastrointestinal side-effects are very common; accumulates in bone and long-term effects in the growing bone are unknown; expensive.
Aluminum hydroxide (or other aluminum containing binders; variable formulations)	0	0	Not applicable	High risk of neurotoxicity if used for long periods; accumulates in bone; not recommended for routine practice, but may be used for short-term ‘rescue’ treatment with close monitoring of aluminum levels.

Oral iron supplements must be given 1–2 h before or after calcium-based binders

Hypocalcaemia, common in untreated CKD, may be seen in patients who present with advanced CKD, in infants who typically have a high Ca requirement, in patients on intensified hemodialysis, and patients receiving a calcimimetic. Treatment with a calcimimetic, similar to a parathyroidectomy, commonly causes “hungry bone syndrome” and often requires more than 200% of the SDI for Ca for prolonged periods to replenish bone Ca stores. A higher Ca intake can be provided through Ca supplementation, together with vitamin D (usually both native and active forms), as well as other sources of Ca such as a high Ca dialysate. Preliminary clinical evidence indicates that in the setting of the hungry bone syndrome exogenous Ca is directed to the bone and not to the vasculature; a high Ca intake is associated with increase in BMD and regression of vascular calcification [98, 99].

Limiting dietary P intake is challenging, and adherence to P binding medications can be poor, especially amongst older children and adolescents; an international registry has shown that 80% of adolescents on peritoneal dialysis have high serum P levels [75]. Limiting the dietary P intake to the lower limit of the SDI may be required if there is either persistent hyperphosphatemia or persistent hyperparathyroidism in the presence of normal Ca levels; a P restriction to the lower limit of the SDI corresponds to the KDOQI recommendation of 80% of DRI [61] for most age groups. It is likely that P binders will be required at this stage, as stringent dietary restriction may be unrealistic and can compromise the intake of protein and other nutrients. As described in section 1, it is important to consider the sources of P and aim to avoid processed foods that are likely to contain inorganic P additives (Table 5), which are almost completely absorbed [100]. Dietary supplements and over-the-counter or prescription medications are hidden sources of P, and may contain P salts in their list of inactive ingredients [101]. The use of P binders can allow a more varied diet and P intake. However, these should not be used as a fixed dose, but should be adjusted to reflect the P content of a meal or snack [102]. For optimal control of P absorption, regular careful review of the use and administration of P binders is essential. Children and their caregivers benefit from regular diet education sessions [103, 104], with help from renal dietitians, as well as doctors and nurses, to understand the complexities of their renal diet [102, 105, 106].

Severe and prolonged dietary P restriction can lead to hypophosphatemic rickets in children. In addition, children on intensified dialysis regimens, including frequent daily or nocturnal dialysis, can have excessive dialysate P losses [107]. While high P losses may be compensated by a higher P diet, some may require P supplementation.

5. Management of the CKD patient with hypercalcemia
  5.1 Acute, *severe* hypercalcemia can be life-threatening and requires rapid medical intervention (grade X, strong).
  5.2 In a child with persistent mild to moderate hypercalcemia, we suggest a stepwise approach with reducing or stopping Ca supplements, Ca-based P-binders, and native and active vitamin D and using lower calcium dialysate. Transient reduction of dietary Ca, without compromising adequate nutrition, may be necessary. Regular reassessment is required, especially when Ca intake is reduced below the SDI (grade C, weak recommendation).

### Evidence and rationale

Acute, severe hypercalcemia can be life-threatening and requires acute medical management; this is beyond the scope of this guideline.

Mild to moderate hypercalcemia can often be effectively addressed by reducing or eliminating intake of Ca supplements, native or active vitamin D, and replacing Ca-based P-binders with Ca-free binders. In addition, the Ca intake from dialysate as well as the Ca intake from other medications (such as potassium binders) must be reviewed and adjusted as best as possible. An investigation into other causes of hypercalcemia may be warranted [108]. A persistently high PTH in the presence of high serum Ca suggests hyperparathyroidism, and a calcimimetic, such as cinacalcet, may be indicated [109–111]. Rarely, high calcium levels in the presence of normal PTH may be seen, such as in immobilized children; in this situation, short-term bisphosphonate therapy may be considered to transiently lower serum Ca. Hypervitaminosis A is also known to cause hypercalcemia, particularly in children on dialysis [112], and must be considered in children with refractory hypercalcaemia. Frequent monitoring of serum Ca, ionized Ca, PTH, and 25-vitamin D levels, and following the trends in levels rather than a single value, is important.

If hypercalcemia persists after reviewing and adjusting medications and dialysate Ca (if appropriate), there is also a need for reduction of dietary sources of Ca and vitamin D, without compromising nutrition. A detailed dietary assessment to determine the key sources of Ca is required (see Table 2). As dairy products usually provide the largest source of Ca in a child’s diet, controlling portion sizes, or finding suitable replacements for cow’s milk, yogurt, and cheese is a primary goal for reducing dietary Ca intake. For infants and young children, a complete low Ca specialized formula (or a formula blend) may be necessary if hypercalcemia is severe and persists. These formulas can be used as a supplementary drink for older children. Plant-derived “milk” products made from oats, soya, almond, coconut, hemp, or similar products that have not been Ca-fortified can be useful for older children, but are often lower in energy and protein. It is advisable not to give rice milk to infants and young children due to its high arsenic content. Limiting intake of other Ca-rich foods may be necessary, including some fish, green-leafy vegetables, beans, nuts, and Ca-fortified foods and drinks. The replacement of tap water with deionized or distilled water also reduces Ca intake. Advice should be individualized, and seeks to minimize compromising the intake of other macro- and micronutrients.

Of note, fortification of foods with vitamin D, in particular dairy products, margarine, and some breakfast cereals, is increasingly common in several countries, and may inadvertently lead to a large cumulative intake of vitamin D, with ensuing hypercalcemia [10]. A careful assessment of food labels to determine if foods are vitamin D fortified is important and parents can be instructed to perform this. The Ca intake from all other sources, including all medications and dialysate, must be carefully recorded and adjusted if appropriate; the reader is referred to other international guidelines for further details [6, 8, 61].

## Results of the Delphi survey

The Delphi survey was sent to 48 pediatric nephrologists and 28 dietitians from 26 countries. Of these, 33 pediatric nephrologists and 15 dietitians returned a completed survey, a 63% overall response rate. The names of all respondents are listed under “Acknowledgments” below.

Of the 13 clinical practice recommendation statements, overall, a 92% consensus was achieved with a “strongly agree or agree” response and a 7.8% “neutral” response. Only 3 statements received a “disagree” response, with the highest “disagree” rate being 5% in response to statement 2. On careful review by the Taskforce team, none of the statements required significant change, but further clarification to the text and tables has been provided as suggested by the respondents.

**Table 9:** Summary of recommendations

	Category	Recommendation	Grade
1	Dietary sources		
	1.1 Dietary sources of Ca	The main dietary sources of Ca for children are milk, milk products, breast milk and manufactured infant formulas. Statutory or voluntary fortification of foods with Ca can increase the contribution from other foods.	Ungraded
	1.1 Dietary sources of P	The main dietary sources of P for children are milk (including milk products, breast milk and manufactured infant formulas), cereals (grains) and cereal products, and meat and meat products. Inorganic P added to some processed foods is readily absorbed and can significantly increase P intake.	Ungraded
2	Assessment of Ca and P intake in healthy children and children with CKD2-5D	We suggest that in healthy children and those with CKD2-5D a diet history of a typical 24-hour period be used to rapidly identify the main sources of Ca and P, including P additives in processed foods. A 3-day prospective diet diary/food intake record may be used when detailed information is required An estimate of the total Ca and P intake should consider contributions from diet, nutritional supplements, dialysate and medications including P binders.	C (weak recommendation)
3	Requirements of Ca and P		
	3.1 Requirements in healthy children	We describe the Ca and P requirements for healthy children as background and justification for estimating the requirements for children with CKD2-5D; specific recommendations for healthy children are outside the scope of this document.	Ungraded
	3.2 Requirements in children with CKD2-5D	3.2.1 We suggest that the diet of children with CKD2-5D should be regularly assessed for total Ca and P content. The contribution of P additives to total P intake cannot be quantified, but dietary sources of P additives should be identified where possible. Frequency of assessment is based on the child’s age, CKD stage and trends in serum Ca, P and PTH.	Ungraded
		3.2.2 We suggest that the total Ca intake from diet and medications, including P binders, should be within the SDI, and be no more than twice the SDI, unless in exceptional circumstances.	C (weak)
		3.2.3 In special circumstances, such as for infants with CKD or those with mineral depleted bone, a higher Ca intake may be considered with careful monitoring.	C (weak)
		3.2.4 We suggest that the dietary P intake of children with CKD should be within the SDI for age, without compromising adequate nutrition	C (weak)
4	Managing the Ca and P requirements in children with CKD2-5D	4.1 We suggest that intake of Ca and P is adjusted to maintain serum Ca and P levels within the age-appropriate normal range, without compromising nutrition. Changes in management should be based on trends of serial results rather than a single result, with integration of serum Ca, P, PTH, alkaline phosphatase and 25-vitamin D levels.	C (weak)
		4.2 We suggest that children with CKD who have hyperphosphatemia or hyperparathyroidism will require further dietary restriction of P, potentially to the lower limit of the SDI, without compromising adequate nutrition. Advice to limit the P contribution from phosphate additives should be given. Use of P binders for further control of serum P and PTH levels is often required, in addition to dietary restriction.	C (weak)
		4.3 We suggest that children with persistent hypocalcaemia or a high PTH may require a Ca intake above 200% of the SDI for calcium for short periods and under close medical supervision. Calcium can be provided through Ca supplementation, together with vitamin D (usually both native and active forms), as well as other sources of Ca such as a high Ca dialysate.	C (weak)
		4.4 We suggest that children with persistent hypophosphatemia should have their dietary P intake increased. P supplements may be necessary in some patients, particularly those on intensified dialysis or with renal wasting of P.	C (weak)
5	Management of hypercalcemia	5.1 Acute, severe hypercalcemia can be life-threatening and requires rapid medical intervention.	X (strong)
		5.2 In a child with persistent mild to moderate hypercalcemia, we suggest a stepwise approach with reducing or stopping Ca supplements, Ca-based P-binders, and native and active vitamin D and using lower calcium dialysate. Transient reduction of dietary Ca, without compromising adequate nutrition, may be necessary. Regular reassessment is required, especially when Ca intake is reduced below the SDI.	C (weak)

### Summary of recommendations

A summary of recommendations is provided in Table 9.

### Research recommendations

We recommend the following areas of study to provide future evidence-based recommendations for Ca and P requirements in children with CKD2-5D:

1. Ca balance studies in children 1–18 years with CKD2-5D to determine the dietary Ca requirements for normal bone mineralization.

2. Ca balance studies to determine the absorption of Ca from Ca carbonate and Ca acetate in children with CKD2-5D who are vitamin D replete.

3. The effectiveness of the 24-h dietary recall as a tool to assess the Ca and phosphate intake in children compared with a 3-day diet diary (semi-quantitative or weighed) or a food frequency questionnaire.

## Electronic supplementary material

ESM 1 (DOCX 359 kb)
