# Supplementary material for: The dietary management of calcium and phosphate in children with CKD stages 2-5 and on dialysis—clinical practice recommendation from the Pediatric Renal Nutrition Taskforce
Source: Pediatr Nephrol. 2019 Oct 30;35(3):501–18. doi: 10.1007/s00467-019-04370-z (PMC6969014; doi:10.1007/s00467-019-04370-z)
Supplement: Supplementary file 1 — (DOCX 359 kb) [file 467_2019_4370_MOESM1_ESM.docx]

**Supplementary Material**

# The dietary management of calcium and phosphate in children with

# CKD stages 2-5 and on dialysis – consensus statement from the

# Pediatric Renal Nutrition Taskforce

**Detailed Methods**

***Overview of the guideline development group composition and task distribution***

Within the PRNT team, three groups were assembled to perform different functions: a core working group (WG), an external advisory group and a review panel. The core group comprised a team of paediatric renal dietitians and Paediatric Nephrologists who are board members of PRNT. An external advisory group included invited dietitians and doctors with expertise in the nutritional management of CKD. The core WG together with the advisory team were responsible for defining the scope of the project, formulating the clinical questions to be addressed, performing a literature review, developing evidence tables, rating the quality of evidence and drafting the guidelines. The chair and all members of the core panel had no relevant conflicts of interest. The review group was comprised of members of the ESPN CKD and Dialysis WGs as well as international experts in the field of renal dietetics and CKD-MBD management. The review group was sent the draft guidance document and all evidence tables and was responsible for reviewing the evidence, confirming the certainty of the evidence and the strength of the statements, and suggesting re-wording of statements if appropriate. Comments received from all members of the review group were collated into a single document and discussed at a meeting of the group. Following an iterative process, a final document was then compiled and circulated to the review group for their opinion.

**Details of the Literature Search**

Medline was searched using the Pubmed interface through to 1^st^ April 2018 using the search terms and strategy detailed in Supplemental Table 1. Limits were pre-set to manuscripts published in the English language, and study design limits were applied. Given the paucity of studies in this field, all publications, including retrospective observational studies, irrespective of patient numbers, have been included. All papers were reviewed by at least two independent reviewers. Data were extracted by at least 2 members, prepared in evidence tables, and reviewed by all members of the PRNT group. Some studies that were outside the remit of the literature review, but contributed important information, have been included in the discussion.

Details on the literature search are described in Supplemental Table 1. Comparative data from international registries describing Ca and P requirements in healthy children are discussed (Table 3).

**Supplemental Table 1.**  **Search terms strategy used in the literature review for MEDLINE and Embase for calcium and phosphate**

**Search terms**

| **1** | **kidney disease** | **renal failure** | **renal insufficiency** | **chronic kidney disease** | **kidney failure** | **kidney injury** | **kidney dysfunction** | **CKD** |
| --- | --- | --- | --- | --- | --- | --- | --- | --- |
|  | **CRF** | **CKF** | **ESRD**  **(and ESKD)** | **ESRF** | **dialysis** | **renal replacement therapy** | **pre dialysis** | **peritoneal dialysis** |
|  | **hemodialysis** | **haemodialysis** | **CAPD** | **APD** |  |  |  |  |
| **2** | **Calcium*** | **Ca*** |  |  |  |  |  |  |
| **3** | **diet** | **dietary** | **nutrition** | **food** | **feed** | **status** | **intake** | **requirements** |
|  | **balance** | **dietary management** | **dietary advice** | **dietary restriction** | **Supplemen-tation** | **dietitian** | **dietician** |  |
|  | Limited to **paediatrics** in Embase (+ ***young adults*** in Medline) | | | | | | | |

*replace calcium or Ca with phosphate or phosphorus for literature search on dietary phosphate studies

**Search methods:**

- Electronic search using Embase and Medline (via OVID online database)

Embase 1974 to 2018 Week 05

MEDLINE(R) without Revisions 1996 to January Week 4 2018

- Reference list from review articles and clinical practice guidelines (including KDOQI & KDIGO)
- Cochrane reviews and the CENTRAL Cochrane registry of controlled trials

**Supplemental Table 2. American Academy of Pediatrics grading matrix**


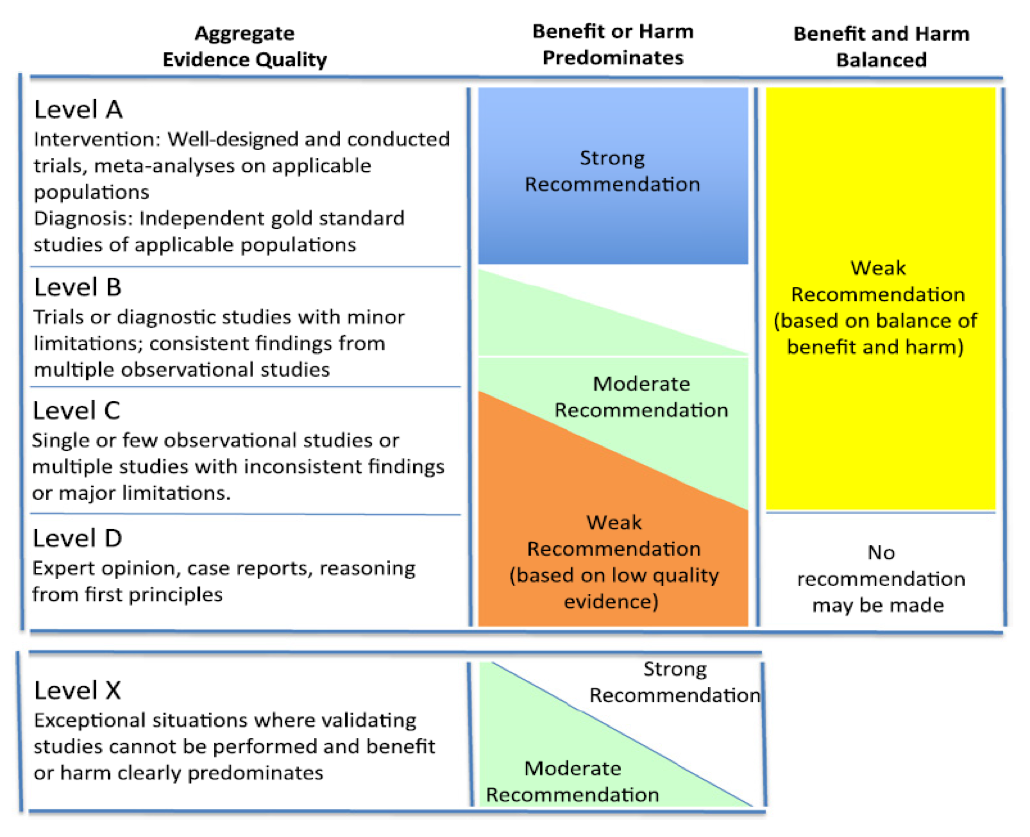


**Supplemental Table 3.**

**Comparison of methods for assessing dietary calcium and phosphate intake**

| **Method** | **Description** | **Advantages** | **Disadvantages** |
| --- | --- | --- | --- |
| **Diet history** | - Parent and child together recall recent usual food and drink intake - Face to face or indirectly by telephone | - Reports usual intake - Minimal participant burden - No literacy skills required - Able to estimate portion size and frequency - Can describe cooking methods and cultural eating habits - Allows classification of nutrient intake into broad categories (low, medium, high) | - Relies on accurate recall - Underreporting or recall bias - Interview can be time consuming - Method not standardised - Trained interviewer required - Interviewer bias - Inaccurate estimation of portion size |
| **Diet diary/food intake record** | - Record of all food and drink consumed over a specified period (3-7 days) - Intake can be weighed or described - Can include product labels - Can either be used to reflect usual intake or include special events - Data is analysed by reference to food composition tables (or data analysis software) | - No recall required - Minimises analysis error if recipes and product labels included - Reproducible - “Gold standard” against which other dietary assessment methods are compared | - High participant burden - Incomplete/selective recording - Literacy skills required - Interpretation bias - Can alter usual food intake |
| **Food frequency questionnaire (FFQ)** | - Report of frequency of consumption of food and drink from a list over a given period - Can be adapted to include portion sizes - Uses closed questions | - Can be tailored to assess specific food groups or nutrients - Provides information on food consumption patterns - Can be adapted to be population/ culture specific - FFQs can be self- completed (depending on complexity) - Simple FFQs impose a low respondent burden - Users can take photos to record portion sizes - Easy to collect and assess - Reliable tool for micronutrient assessment | - Relies on accurate recall - Time consuming depending on number of food items included - Literacy skills required |
